# Analysis of the Features Important for the Effectiveness of Physical Activity–Related Apps for Recreational Sports: Expert Panel Approach

**DOI:** 10.2196/mhealth.9459

**Published:** 2018-06-18

**Authors:** Joan Dallinga, Mark Janssen, Jet van der Werf, Ruben Walravens, Steven Vos, Marije Deutekom

**Affiliations:** ^1^ Faculty of Sports and Nutrition Amsterdam University of Applied Sciences Amsterdam Netherlands; ^2^ Faculty of Health, Sports and Social Work Inholland University of Applied Sciences Haarlem Netherlands; ^3^ School of Sport Studies Fontys University of Applied Sciences Eindhoven Netherlands; ^4^ Department of Industrial Design Eindhoven University of Technology Eindhoven Netherlands

**Keywords:** mobile applications, exercise, healthy lifestyle, mHealth, measures, health behavior, features

## Abstract

**Background:**

A large number of people participate in individual or unorganized sports on a recreational level. Furthermore, many participants drop out because of injury or lowered motivation. Potentially, physical activity–related apps could motivate people during sport participation and help them to follow and maintain a healthy active lifestyle. It remains unclear what the quality of running, cycling, and walking apps is and how it can be assessed. Quality of these apps was defined as having a positive influence on participation in recreational sports. This information will show which features need to be assessed when rating physical activity–related app quality.

**Objective:**

The aim of this study was to identify expert perception on which features are important for the effectiveness of physical activity–related apps for participation in individual, recreational sports.

**Methods:**

Data were gathered via an expert panel approach using the nominal group technique. Two expert panels were organized to identify and rank app features relevant for sport participation. Experts were researchers or professionals in the field of industrial design and information technology (technology expert panel) and in the field of behavior change, health, and human movement sciences who had affinity with physical activity–related apps (health science expert panel). Of the 24 experts who were approached, 11 (46%) agreed to participate. Each panel session consisted of three consultation rounds. The 10 most important features per expert were collected. We calculated the frequency of the top 10 features and the mean importance score per feature (0-100). The sessions were taped and transcribed verbatim; a thematic analysis was conducted on the qualitative data.

**Results:**

In the technology expert panel, applied feedback and feedforward (91.3) and fun (91.3) were found most important (scale 0-100). Together with flexibility and look and feel, these features were mentioned most often (all n=4 [number of experts]; importance scores=41.3 and 43.8, respectively). The experts in the health science expert panels a and b found instructional feedback (95.0), motivating or challenging (95.0), peer rating and use (92.0), motivating feedback (91.3), and monitoring or statistics (91.0) most important. Most often ranked features were monitoring or statistics, motivating feedback, works good technically, tailoring starting point, fun, usability anticipating or context awareness, and privacy (all n=3-4 [number of experts]; importance scores=16.7-95.0). The qualitative analysis resulted in four overarching themes: (1) combination behavior change, technical, and design features needed; (2) extended feedback and tailoring is advised; (3) theoretical or evidence base as standard; and (4) entry requirements related to app use.

**Conclusions:**

The results show that a variety of features, including design, technical, and behavior change, are considered important for the effectiveness of physical activity–related apps by experts from different fields of expertise. These insights may assist in the development of an improved app rating scale.

## Introduction

### Recreational Sport Participation

Starting with and maintaining physical activity (PA) is a challenge for many citizens. We see that in the United States and Europe, physical inactivity and sedentary behavior are increasing, causing health-related problems such as decreased quality of life and increase in health care costs [1]. Potentially, participation in sports can contribute to a more healthy lifestyle [2-6]. However, participation rates are also quite low, with 59% of European citizens exercising or playing a sport less than once a week [7]. In the Netherlands, the situation is slightly more positive, with 44% of Dutch citizens participating in sports less than once a week or never [8]. Of the citizens that participate in recreational sports in the United States and Europe, a large number of people participate in individual or unorganized sports (eg, running and cycling) [9-13]. In the Netherlands, the participation in recreational individual sports such as running, cycling, walking, and fitness is increasing as well [8]. A large part of these participants are beginners or less experienced. These individual sports are often practiced in lighter nonclub-organized settings (leisure time sport participation that allows for a flexible experience) or individually [14]. In the latter, there exist no or limited support and guidance of a trainer or coach. Therefore, these individual athletes are at risk of injuries or loss of motivation and hence dropping out and therefore decreasing PA [15]. Substantial guidance is necessary to prevent injuries and to stay motivated to participate in sports, especially among beginner and less experienced participants [16,17].

### Potential of Physical Activity–Related Apps

Potentially PA–related apps could motivate these people during sport participation and help them to follow and maintain a healthy and active lifestyle. Mobile health (mHealth)–related apps are popular; in 2016, the app stores displayed 105,000 (Google Play) and 126,000 (Apple Play Store) mHealth-related apps in health and fitness and medical categories [18]. In recent years, a large number of PA–related apps have been developed for people in individual sports, and every day, new apps are being launched in the app stores [19,20]. Previous research shows that approximately 50% to 75% of (event) runners use a running app [15,21]. Cycling and walking apps are gaining in popularity as well. For instance, Strava (an app for running and cycling) has millions of users and the number of users increases each month. [22-24]. In contrast to the published data available on the use of running and cycling apps, little is known about the use of walking apps.

These running, cycling, or walking apps provide possibilities to support people in participation in exercise and sports (such as monitoring activities, setting goals, and comparing your results to others) [25,26]. However, the question is whether the quality of currently available apps is sufficient to support recreational sport participants. An analysis of the quality of apps and knowledge about which app features matter the most is necessary to determine whether apps have added value.

### Assessment of Physical Activity–Related Apps

The quality of PA–related apps has been evaluated in various manners in previous research. Some studies have examined if and how many behavior change techniques (BCT’s) are applied in current health– or PA–related apps by using an app taxonomy of Abraham and Michie [26-30]. Results showed that only a small amount of BCT’s (mean number of 3.7-8 BCT’s) are applied in PA or healthy nutrition apps [26,28,30]*.* Content analyses of apps also showed that the evidence base of currently available health and fitness apps is limited [31-33]. A recent study evaluated if and how gamification was used in health and fitness apps [34]. They showed that gamification features were often used in popular apps; however, low adherence to professional guidelines or industry standard for gaming was found [34]. Other app rating scales have been developed as well, such as the Mobile App Rating Scale (MARS) and an app rating scale for exercise apps [35,36]. The MARS was developed for classifying and assessing the quality of mHealth apps [35]. In general, moderate quality scores were found for mental health and wellbeing apps and weight management apps [35,37]. The app rating scale for exercise apps developed by Guo et al (2017) was based on exercise prescriptions developed by the American College of Sports Medicine (ACSM) for aerobic exercise, strength and resistance, and flexibility [36]. On the basis of this scale, low scores (maximum 35 out of 70 points) were found for the tested exercise apps [36]. Another method to evaluate the quality of PA–related apps is by assessing technical features or design. The mHealth taxonomy of Olla and Shimskey examines features such as data management, user interface, and device type [38]. The MARS evaluated technical features as well, such as having an app community and containing data protection using a password [35]. However, these technical features were not included in the quality score of the app.

In summary, a variety of app features have been examined in current app rating scales, including design, technical, and behavior change features. In some of these rating scales (eg, MARS and taxonomy of Abraham and Michie) [29,35], all app features are considered evenly important, whereas the rating scale developed by Guo et al applied a weighting to the items [36]. The time allocated to different components (aerobic exercise, strength and resistance, and flexibility) of a standard exercise program for health and fitness (ACSM guidelines) was used to weigh the items [36].

### Problem Statement

This study is innovative in two ways: the incorporation of experts’ opinions (instead of based on literature or theories on behavior change) and the assessment of the importance of features (instead of only the presence of features). It remains unclear how the quality of running, cycling, and walking apps, defined as having a positive influence on participation in recreational sports, can be assessed. We do not know if some app features may be more important than others for participation in recreational sports and if a weighing should be applied. In addition, there is currently no PA–related app rating scale that scores design, technical, and behavior features. Currently available app rating scales are based on literature or theories on behavior change but do not take into account the opinion of experts regarding the importance of app features. In this study, experts were defined as researchers or professionals in the field of behavior change, psychology, health, and human movement sciences, as well as industrial designers and information technologists. Their knowledge of and experience with design and evaluation of PA–related apps is deemed to be very valuable. The obtained additional information regarding the rating of features can be used in the development of an improved PA–related app check list.

### Objective

Therefore, the aim was to identify expert perception on which features are important for the effectiveness of PA–related apps for participation in individual, recreational sports.

## Methods

### Design

The data were gathered via an expert panel approach in which the nominal group technique (NGT) was used [39]. Two expert panels were organized to identify and rank app features relevant for effectiveness of PA–related apps for participation in individual, recreational sports. This NGT was chosen for this study as it provides the possibility to identify problems and gain more insight in a topic by quantifying opinions of participants in a democratic way [39,40]*.* In addition, the NGT includes a structured group process and can be used to generate and rank ideas for group discussion, to reach consensus, and to engage group members to solve a problem [41]. The NGT was proven evenly effective as other methods in terms of accuracy, idea selection, and satisfaction with the process, such as face-to-face meetings, Delphi method, and interactive groups [42,43]. Moreover, a previous study showed that it was an effective and efficient tool to generate ideas and to develop consensus in a group of experts [44]. Small and rather homogeneous groups are preferred in using NGT [45].

### Participants

A total of 12 experts for each panel (24 experts in total) were recruited and approached, taking into account dropout, among others, because of time constraints. Convenience sampling was used to recruit the experts. These experts were selected based on their experience, expertise, and perception concerning PA–related apps [39]. All experts needed to have a Master’s degree. Two types of experts were selected for these two panels. Inclusion criteria for the first group (technology expert panel) included scientific background in information and communication technology (ICT), service design, industrial design, or research through design (or other comparable fields). Inclusion criteria for the second group (health science expert panel) included (1) Scientific background in behavioral, psychological, health, or human movement sciences (or other comparable fields) *or* professional experience in these domains and (2) Research *or* professional experience (at least 3 years) with PA–related apps. This way, knowledge and expertise from different disciplines was collected. This study was part of a larger research project called “An app for everyone?!” The aim of this project was to determine which (popular) sport app fits which type of user or professional based on their goals and wishes. If the selected experts were already involved as partner in this research project, they were excluded. All experts signed an informed consent before participating in the expert panels.

Of the 24 approached experts, 11 (46%) were able to attend the expert panel sessions. Four experts were included in the first session and seven in the second group session. Due to time restrictions, the other 13 experts were not available on the scheduled sessions. Still, we were able to include all relevant expertise in the panels.

Table 1 presents the characteristics of the experts who participated in the panels. For the NGT, the health science expert panel was divided into two subgroups (a group of three [health science expert panel A] and a group of four experts [health science expert panel B]), to make sure that all experts had enough time to express their thoughts and that there was enough time for discussion [45].

### Procedure

First, the selected experts were contacted via email to participate in the NGT. All experts who agreed to participate received an email with additional information about the purpose and procedure of the study. The first expert panel (technology expert panel) was organized on October 18, 2016 and was facilitated and observed by two of the authors (RW and JD). Subsequently, the second expert panel (health science expert panel) interview was organized on October 31, 2016 and was facilitated by two of the authors (JD and JvdW). The sessions were organized at a location that was most convenient for the experts (Eindhoven and Amsterdam, The Netherlands). To increase the reliability and validity of the results, the moderators followed the same protocol, and one moderator attended both sessions.

**Figure 1:**
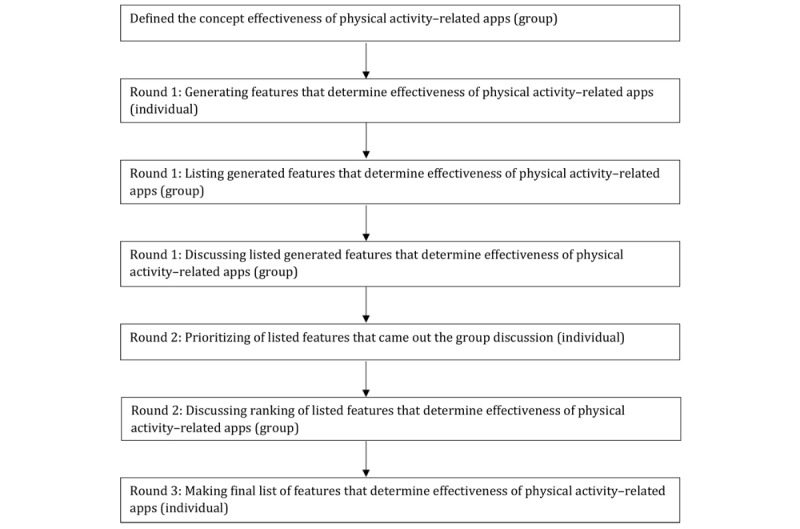
Three rounds of the nominal group technique.

In alignment with the NGT, each session consisted of three consultation rounds [46]. In these three rounds, the goal was to rank and prioritize PA–related app features (Figure 1). To facilitate interaction, name tags were placed in front of the experts, and the experts were positioned in a half-circle. The moderator facilitated the discussion, provided instructions about the assignments, and ensured that all experts had an equal say. If necessary, the moderator asked for clarification of the answers provided by the experts.

**Table 1:** Expert characteristics.

Characteristics		Technology expert panel (n)	Health science expert panel A (n)	Health science expert panel B (n)
**Sex**				
	Male	4	2	0
	Female	0	1	4
**Expertise**				
	Behavior change	0	1	2
	Human movement sciences (injury prevention or monitoring)	0	1	1
	Health sciences	0	1	0
	Persuasive technology	0	0	1
	ICT^a^ service design	2	0	0
	Industrial design	2	0	0
**Degree**				
	MSc	2	3	2
	PhD	2	0	2

^a^ICT: information and communication technology.

In a short introduction, the moderator explained the framework of the session. The moderator asked the experts to focus on running, cycling, and walking apps for recreational athletes, with a goal to start and maintain sports participation. After this introduction, all participants introduced themselves and explained their experience with PA–related apps. Subsequently, the moderator informed the experts about the purpose of the research project and the protocol. To set a framework for the assignments in the sessions, we asked the experts to define the concept “effectiveness of apps.” The experts discussed in their own sessions their shared idea about what effectiveness meant to them (social construction).

In the first round, the experts were asked to individually list all app features that they found necessary for effectiveness of PA–related apps for sport participation (Multimedia Appendix 1). After that, these features were collected, explained, and listed on a white board. In the second round, the experts were asked to individually rank the 10 features they found most important (Multimedia Appendix 2). Subsequently, these rankings were collected, presented on a screen, and discussed groupwise. In the last round, the experts individually made a final list of their 10 most important features (Multimedia Appendix 3). In addition, they were also asked to appoint a score to each feature (0-100), to indicate importance. The duration of both expert panel sessions was 2 hours.

### Data Analysis

#### Nominal Group Ranking

On the basis of the third round, the 10 most important features per expert were collected. The features generated by the expert panel sessions were combined into one list per panel. We calculated the frequency of the features in the top 10, as well as the mean importance score per feature. Differences between groups were not calculated because of small sample size.

#### Qualitative Analysis

The sessions were audiotaped and videotaped and transcribed verbatim. On the basis of these transcripts, a list of features generated by each group and their definitions was created. The transcripts were read and reread by one of the authors (JD). After that, a thematic analysis was conducted on the qualitative data from the expert panels. This thematic analysis focused on the answers that illustrated and supported the experts’ ranking choices. The coding was performed manually based on a coding framework that was developed inductively. This coding frame was discussed and checked by a coinvestigator (JvdW) who was a moderator as well (investigator triangulation) [47].

## Results

### Structure of Results

The results from this study are presented in three sections. The first section shows how the experts defined the concept effectiveness of PA–related apps as a starting point of the discussion. The second section presents the various features that were ranked and their importance. The third section provides some of the overarching themes that were extracted from the panel sessions. These themes can be considered important areas to address in the development of a new app rating scale.

### Definition of Effectiveness

At the start of the panel sessions, the experts defined the concept effectiveness of PA–related apps to delineate the topic.

In the first expert panel, the experts agreed that an app was effective if a (safe, sustainable and healthy) change of behavior was established. Experts from the second panel (health science expert panel A and health science expert panel B) agreed on that and added that an app was effective if it could change behavior determinants such as knowledge, attitude, risk perception, and awareness to influence behavior on the long term.

### Nominal Group Ranking

In total, 51 features were collected in round one. After selecting, prioritizing, and discussing these features in round two and three, 25 features remained and were ranked by the experts in both expert panels. Table 2 shows for each panel frequency of the features in the top 10, as well as the mean importance score per feature (on a scale of 0-100). The total frequency of individually ranked features ranged from 1 to 9.

The features that were perceived as most important by the technology expert panel (with industrial designers and information technologists) were applied feedback and feedforward (anticipating on future behavior or goals; 91.3) and fun (91.3). Besides those two features, look and feel and flexibility were also mentioned most often (all n=4 [n denotes the number of experts]). The importance scores of these two features were considerably lower (43.8 and 41.3, respectively). The experts in the health science expert panel A (behavior change and human movement sciences) found instructional feedback (95.0), motivating feedback (91.3), and monitoring or statistics (90.0) most important. The features that were ranked most often (number of experts=4) were monitoring or statistics, motivating feedback, technically properly working, tailoring starting point, fun or pleasure, and usability. The importance scores of these features were high as well (82.0-95.8).

Experts in the health science expert panel B found motivating or challenging (95.0), monitoring or statistics (95.0), and peer rating and use (92.0) most important. Usability, anticipating or context awareness, and privacy were ranked by all experts in this subpanel, with importance scores ranging from 16.7 to 85.0.

### Qualitative Analysis

During the panel sessions, the experts elaborated on the features they ranked and explained why they found them important. This section outlines the overarching themes that were found.

Each theme is discussed below and illustrated with quotes of the experts. In Multimedia Appendix 4, a detailed version of the qualitative analysis is presented, and in Multimedia Appendix 5, the corresponding coding scheme is provided.

#### Combination Behavior Change, Technical, and Design Features Needed

In line with the expertise of the expert panels, features for behavior change as well as technical and design features were considered as important for effectiveness of PA–related apps. For instance, in the technology expert panel next to technical and design features, applied feedback, fun, rewards, and context awareness were ranked in the top 10. In addition, in health science expert panel A and health science expert panel B, next to behavior change features, reliability, usability, works good technically, and visibility were ranked in the top 10. Experts in technology expert panel indicated that, for these features, in general, domain specific knowledge is required, as illustrated in the following quote:

Basically applied feedback includes knowledge of sports, motivational support and quality of coaching, and depends on the intended application.

Technology expert panel, expert in industrial design

**Table 2:** Features ranked by experts in round 3 (based on top 10 ranking).

Feature	Technology expert panel		Health science expert panel A		Health science expert panel B		All experts	
	Mean importance score^a^	Frequency	Mean importance score^a^	Frequency	Mean importance score^a^	Frequency	Mean importance score^a^	Frequency
Instructional feedback	—^b^	—	95.0	2	—	—	95.0	2
Motivating or challenging	—	—	—	—	95.0	1	95.0	1
Peer rating and use	—	—	—	—	92.0	1	92.0	1
Applied feedback and forward	91.3	4	—	—	—	—	91.3	4
Motivating feedback	—	—	91.3	4	—	—	91.3	4
Monitor or statistics	—	—	90.0	4	95.0	1	91.0	5
Stability	90.0	1	—	—	—	—	90.0	1
Engagement	—	—	—	—	87.5	2	87.5	2
Technically properly working	—	—	87.5	4	—	—	87.5	4
Tailoring starting point	—	—	85.0	4	—	—	85.0	4
Continues tailoring	—	—	85.0	3	—	—	85.0	3
Usability	60.0	1	87.5	4	85.0	3	83.1	8
Fun or pleasure	91.3	4	73.8	4	85.0	1	82.8	9
Rewards	65.0	2	—	—	95.0	1	75.0	3
Reliability	—	—	80.0	1	70.0	1	75.0	2
Theoretical (scientific) base or evidence + BCT’s^c^	—	—	—	—	75.0	2	75.0	2
Check on health	—	—	73.3	3	—	—	73.3	3
Visibility or exposure or reputation	—	—	—	—	72.5	2	72.5	2
Social	80.0	2	55.0	2	90.0	1	72.0	5
Coaching styles	—	—	—	—	70.0	1	70.0	1
Tailoring content that cannot be changed	—	—	70.0	1	—	—	70.0	1
Connectivity	70.0	2	—	—	—	—	70.0	2
Costs	—	—	70.0	1	—	—	70.0	1
Fit to user or everyday life or tailoring	71.7	3	—	—	60.0	1	68.8	4
Sustainable training plan	—	—	60.0	1	—	—	60.0	1
Anticipating or context awareness	35.0	2	73.0	2	48.3	3	51.9	7
General information healthy behavior	—	—	50.0	1	—	—	50.0	1
Increase awareness	50.0	2	—	—	—	—	50.0	2
Flexibility or adjustable or adaptive	41.3	4	—	—	60.0	1	45.0	5
Look and feel	43.8	4	—	—	—	—	43.8	4
Portability	40.0	1	—	—	—	—	40.0	1
Privacy	—	—	—	—	16.7	3	16.7	3

^a^On a scale from 0 to 100.

^b^Experts from respective panel did not rank feature.

^c^BCT: behavior change technique.

#### Extended Feedback and Tailoring Is Advised

Experts emphasized that a feedback option, as well as extended tailoring, needs to be integrated in a PA–related app. Several feedback options were suggested, such as motivational feedback (positive framing) and instructional feedback (health science expert panel A), as illustrated in the following quote:

You should be approached in a positive way, even if you haven’t done anything that day.

Health science expert panel B, expert in persuasive technology

Coaching styles in a PA–related app matter as well and should be tailored to the individual athlete (health science expert panel B). Tailoring in general can be applied in several ways: at the moment a person starts using the app or continued tailoring during the whole process of using an app. This tailoring should be aligned with the current level of health, knowledge, functioning, personal goals, competitiveness, PA, and personal characteristics. One expert stated the following:

To me, it is important that the tailoring should fluctuate with one’s life.

Health science expert panel B, expert in behavior change

Another element of tailoring is the flexibility of the app, in other words being able to adjust the app and adaptivity of the app. One expert stated the following:

For instance, if your running performance improves, the app should develop as well.

Technology expert panel, expert in industrial design

One step further would be that the app should anticipate on the user. For instance, by accounting for schedules and location. This feature was described as context awareness and was discussed in all panel sessions. One expert stated the following:

That you reckon with someone’s context. That it can account for the fact that not all things go as planned.

Health science expert panel B, expert in persuasive technology

As an example, a recommender system was described. A recommender system is a machine learning, information-retrieval software tool that predicts what a user may or may not like or need [48]. It can provide suggestions based on these predictions.

#### Theoretical or Evidence Base Is the Standard

Two experts from health science expert panel A and health science expert panel B indicated that in general, a theoretical or evidence base was important for the effectiveness of PA–related apps. Some examples of BCT’s were briefly mentioned, including self-regulation, goal setting, overview of results, tailoring, monitoring, context awareness, nudging, and self-learning. Other BCT’s were discussed more in detail in the panels, such as fun, social component, monitoring, rewarding, feedback or coaching, tailoring, and information about healthy and safe sport participation. Besides BCT’s, other potential theories were mentioned, such as technological- and medical-based theories or engagement theories for the development of apps, as illustrated in the following quote:

There are many other theories for building apps and you could take these into account as well. It is not only about behavior change. The app could be built based on a technical or medical view or engagement theories as well.

Health science expert panel A, expert in health sciences

One expert in behavior change highlighted that an evidence and a theoretical base are two different things. An app can be based on a theoretical model but can lack an evidence base. The transtheoretical model was used as an example. One expert stated the following:

For instance, the Transtheoretical model, which is a typical theoretical foundation. If you look at the empirical evidence, it is not that good.

Health science expert panel A, expert in behavior change

The same expert indicated that an expert rating of the PA–related app could also be interpreted as an evidence base.

#### Entry Requirements Related to App Use

These are minimum conditions that support the use of the app. Examples are looks and usability, image of the app, and other requirements such as privacy and costs of the app.

At first, form, language, design, tone, and interaction were described as important entry requirements for an effective app. Second, usability was found important. It was defined in several ways and was related to functioning and simplicity of the app. One expert stated the following:

Does the app do what you expect from it and do specific functions work properly. It shouldn’t be too complex and searching for functions should not take too much time.

Health science expert panel B, expert in injury prevention and monitoring

Furthermore, according to an expert, usability of an app could be related to motivation to be active; the technical application and design of push notifications directed at motivating the app-user matter. He stated the following:

Usability, or ease of use, does it motivate you? Think about a push notification if you haven’t done a task. This is a more functional application to motivate you. Not so much the knowledge and content is important, but also the technical application of a push message.

Technology expert panel, expert in ICT service design

Stability, reliability, and robustness of the app were related to usability as well, as illustrated in the following quote:

So actually it is about how much you trust the app.

Technology expert panel, expert in ICT service design

A third requirement was that the app should function properly, without bugs. Moreover, being able to connect the app to other tools (such as an online platform, activity tracker, or smartwatch) or being able to exchange data between platforms (ie, portability) contributes to the usability of the app.

Experts from the health science expert panel B noticed that the image of the app may contribute to the effectiveness of a PA–related app. The image of the app depended on the reliability (credibility and properly functioning measurements and feedback), visibility, exposure, and popularity of the app. Exposure was described as brand awareness. One expert stated the following:

If there are a thousand apps in the app stores, you should be able to look at a screen shot and know “this is what I was looking for”...This has to do with exposure and marketing.

Health science expert panel A, expert in behavior change

Two other entry requirements were discussed: costs of the app and privacy. Some experts thought that people would be more willing to download an app if it is free. However, according to some of the experts, you could see it as an investment as well. When you invest money in an app, then you may be more motivated to continue using it and potentially stay active as well. Two experts thought the price-quality ratio was more important for the effectiveness of an app, than the price only, as illustrated in the following quote:

The price does not determine the quality! That is not how I experience it.

Technology expert panel, expert in ICT service design

Privacy was described as an upcoming topic. In other words, what do you have to say about your data, but additionally, what do app owners do with the collected data of users. The experts indicated that knowing how the privacy of your data is secured is an important entry requirement.

## Discussion

### Principal Findings

In this study, we conducted expert panels using the NGT to determine the perception of experts on which features are important for effectiveness of PA–related apps for participation in individual, recreational sports. A total of 25 features were ranked. Applied feedback and feedforward and fun were the most important features for experts in the field of industrial designers and information technologists. Instructional feedback, motivating feedback, motivating or challenging and monitor or statistics, and peer rating and use were the most important features for experts on behavioral, health, and human movement sciences. The features monitoring or statistics, motivating feedback, technically properly working, tailoring starting point, fun or pleasure, usability, flexibility, look and feel, anticipating or context awareness, and privacy were frequently ranked in the top 10 as well. In line with the expertise of the two expert panels, features for behavior change as well as technical and design features were collected.

A qualitative analysis of the reasons behind the expert’s choices showed four overarching themes: (1) combination behavior change, technical, and design features needed, (2) extended feedback and tailoring is advised, (3) theoretical or evidence base is the standard, and (4) entry requirements related to app use.

### Comparison With Prior Work

The experts found a theoretical framework important; they ranked several features that were previously defined as BCT’s in the taxonomy of Abraham and Michie [29]. Some of the ranked features were included in the MARS as well, such as engagement, usability, customization, and aesthetics [35]. However, based on the results of this study, more advanced features seem necessary to support sport participation. For instance, tailoring was an important feature with several subdomains, such as tailoring on start level, continued tailoring, adaptivity, and flexibility of the app. In contrast, the MARS only includes one question about customization [35]. A recent review suggested that a tailored approach in PA–related apps may increase their efficacy [49], which is in line with the present results. Furthermore, Op den Akker et al (2014) proposed a framework for tailoring of real-time PA coach systems [50]. The key concepts of this framework were feedback, interhuman interaction, adaptation, user targeting, goal setting, context awareness, and self-learning, which corresponds partly to our findings. Most of these concepts, such as feedback, adaptation, goal setting, context awareness, and self-learning were mentioned by the experts. In summary, the features the experts in this study described as important were in line with previous studies; however, subcategories of these features were ranked that were not perceived as evenly important. Potentially, a more detailed analysis of app subfeatures is necessary to determine the quality of PA–related apps for individual, recreational sport participants.

One expert highlighted that it is important to pay attention to the health aspects and safe sport participation. This was supported by the other experts (although not ranked in top 10). This matches with one of the BCT’s (provide information about behavior-health link) as defined by Abraham and Michie [29]. The MARS offers an option to rate the potential impact of the app on the user’s knowledge, attitudes, and intentions related to the healthy behavior [35]*.* However, according to the experts, these features seem essential and therefore, need to be included in the assessment of the quality of apps.

The experts rated and prioritized several types of features, including design, technical, and behavior change features. Interestingly, they also emphasized that domain specific knowledge should be integrated into PA–related apps. Technical features such as stability, portability, and connectivity were not included in the MARS [35]. In the MARS, some technical elements can be scored as yes or no in a checkbox. This does not indicate the degree in which this feature is integrated or designed. In line with our results, a previous study proposed that technical modalities in apps need to be considered in a taxonomy for mHealth apps [38]. Examples are the device type (which device is needed), interface (user-friendly interface), operating system type (eg, Android or iPhone operating system, iOS, Apple Inc), and features (audio, video, email). In summary, current app ranking tools often focus mostly on one domain [29,36]. For instance, the MARS focuses mostly on behavior change [35], whereas the taxonomy of Olla and Shimskey focuses mostly on technical features [38]. We suggest that a multidisciplinary approach is suitable when examining the quality of PA–related apps. Behavior change, design, and technical features need to be assessed in a PA–related app rating scale.

The results of this study indicate that experts find some features from the top 10 more important than others. For instance, instructional feedback was ranked most important and privacy as least important in the health science expert panel. This may suggest that an app rating scale should apply a weighing of the items. Additionally, the qualitative analysis also showed that there are some entry requirements for the effectiveness of a PA–related app. Without these features, the app probably will not be used. Therefore, we suggest that an app rating scale should contain a specific subsection in which entry requirements should be scored.

Interestingly, the experts indicated that more advanced features are needed to support sport participation. However, we need to keep in mind that the PA–related apps available in the app stores often lack a theoretical or evidence base and do not include advanced features. For instance, to the best knowledge of the authors, current PA–related apps do not take into account more advanced forms of tailoring, such as context awareness or tailoring on starting level and continued tailoring as suggested in this study. This highlights a gap between desired features in an optimal PA–related app and the features that are included in PA–related apps at this moment.

### Strengths and Limitations

A strength of our study was that we included experts from different expertise in the panels. Our study is subject to some limitations as well. First, several potential experts (2x12) were selected and invited for the sessions; however, many of them were not able to attend the session because of practical matters. Therefore, the number of experts was low. This may have decreased the generalizability of the results. Next, a convenience sample of experts were selected, as the experts needed to be able to travel to one of the two locations. This selection method could have resulted in selection bias, which could imply that we may have missed some important perspectives. Still, we were able to select experts with relevant experience and knowledge of development and evaluation of PA–related apps. Therefore, we think that these 11 participants provide a quite good representation.

We selected experts based on their scientific and professional expertise and therefore, think the experts had knowledge about current literature on PA–related apps. However, it is still possible that the experts believe that certain features are important for effectiveness of PA–related apps that in fact objective evidence may show are not effective. In the development of an improved PA–related rating scale, it is therefore recommended to combine the results of this study on expert opinions about important features with a literature review.

### Conclusions

Taken together, the results show that experts from different fields of expertise think that a variety of features, including design, technical, and behavior change, are considered as important for the effectiveness of PA–related apps for sport participation. These results may assist in the development of an improved app rating scale for these apps that can indicate the quality. In other words, which PA–related apps could motivate (beginning) individual recreational sport practitioners during sport participation and support or help them with a healthy active lifestyle. On the basis of the results of this study, we recommend for the development of an improved PA–related app rating scale:

- To rate as well behavior change features as design and technical features
- Include assessment of theoretical or evidence base of the app
- A more detailed analysis of app subfeatures, for instance tailoring on start level, continued tailoring, adaptivity, and flexibility of the app
- Rate if the app informs about healthy and safe sports participation
- Rate entry requirements such as usability, bugs in the app, and image

The results of this paper are relevant for PA–related app designers as well. On the basis of this study, our advice is to work together with experts from different domains in the development of PA–related apps, take into account factors related to app use and app engagement (entry requirements), and make sure the app has a theoretical or evidence base. Furthermore, this paper indicates which features may be important to include in a PA–related app.

## Appendix

Multimedia Appendix 1 Expert panel form A.

Multimedia Appendix 2 Expert panel form B.

Multimedia Appendix 3 Expert panel form C.

Multimedia Appendix 4 Extended version of the qualitative analysis.

Multimedia Appendix 5 Coding scheme.

## Abbreviations

ACSM: American College of Sports Medicine
BCT: behavior change technique
ICT: information and communication technology
MARS: Mobile App Rating Scale
mHealth: mobile health
NGT: nominal group technique
PA: physical activity
