# Supplementary figures and images for: Analysis of the Features Important for the Effectiveness of Physical Activity–Related Apps for Recreational Sports: Expert Panel Approach
Source: JMIR Mhealth Uhealth. 2018 Jun 18;6(6):e143. doi: 10.2196/mhealth.9459 (PMC6028765; doi:10.2196/mhealth.9459)

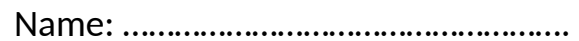[illegible]

Supplement: Multimedia Appendix 1 [file mhealth_v6i6e143_app1.pdf]
