# Supplementary material for: Analysis of the Features Important for the Effectiveness of Physical Activity–Related Apps for Recreational Sports: Expert Panel Approach
Source: JMIR Mhealth Uhealth. 2018 Jun 18;6(6):e143. doi: 10.2196/mhealth.9459 (PMC6028765; doi:10.2196/mhealth.9459)
Supplement: Multimedia Appendix 2 [file mhealth_v6i6e143_app2.pdf]

Multimedia Appendix 2

Expertpanel form B

An app for everyone?!

Amsterdam - 31 October 2016 Name: ……………………………………………..

Which 10 factors determine, according to you, the effectiveness of apps the most? (Ranking 1 is most important and 10 the least important)	
**Ranking 1**	
**Ranking 2**	
**Ranking 3**	
**Ranking 4**	
**Ranking 5**	
**Ranking 6**	
**Ranking 7**	
**Ranking 8**	
**Ranking 9**	
**Ranking 10**	
