# Supplementary material for: Analysis of the Features Important for the Effectiveness of Physical Activity–Related Apps for Recreational Sports: Expert Panel Approach
Source: JMIR Mhealth Uhealth. 2018 Jun 18;6(6):e143. doi: 10.2196/mhealth.9459 (PMC6028765; doi:10.2196/mhealth.9459)
Supplement: Multimedia Appendix 3 [file mhealth_v6i6e143_app3.pdf]

Multimedia Appendix 3

Expertpanel form C

An app for everyone?!

Amsterdam - 31 October 2016 Name: ……………………………………………..

Which 10 factors determine, according to you, effectiveness of apps? (Give each factor a score between 0-100 to score the importance of the factor in relation to the proposed question)		
	Factor	Score
**1**		
**2**		
**3**		
**4**		
**5**		
**6**		
**7**		
**8**		
**9**		
**10**		
