# Supplementary material for: Analysis of the Features Important for the Effectiveness of Physical Activity–Related Apps for Recreational Sports: Expert Panel Approach
Source: JMIR Mhealth Uhealth. 2018 Jun 18;6(6):e143. doi: 10.2196/mhealth.9459 (PMC6028765; doi:10.2196/mhealth.9459)
Supplement: Multimedia Appendix 4 [file mhealth_v6i6e143_app4.pdf]

Multimedia Appendix 4 extended version qualitative analysis

Based on the qualitative analysis, we identified two main themes of features from the expert panels, (1) which were features related to (behavior change) theories and (2) entry requirements for apps. These minimum entry requirements comprised features that should be included in the app to function properly. The two main themes were divided into several sub themes (Multimedia Appendix 5), and each theme is discussed separately. Below these subthemes are described for each panel.

1. Features related to (behavior change) theories

Theoretical or evidence base

Two experts from health science expert panel indicated that in general, a theoretical or evidence base was important for the effectiveness of physical activity-related apps. Some examples of BCT's were briefly mentioned, including self-regulation, goal setting, overview of results, tailoring, monitoring, context awareness, nudging, and self-learning. Besides BCT's, an expert in health sciences mentioned other potential theories, such as technological and medical based theories or engagement theories for the development of apps as well.

"There are many other theories for building apps and you could take these into account as well. It is not only about behavior change. The app could be built based on a technical or medical view or engagement theories as well." (health science expert panel, expert in health sciences)

One expert in behavior change highlighted that an evidence base and a theoretical base are two different things. An app can be based on a theoretical model, but can lack an evidence base. The trans theoretical model was used as an example. This expert said:

"For instance, the trans theoretical model, which is a typical theoretical foundation. If you look at the empirical evidence, it is not that good" (health science expert panel, expert in behavior change)

The same expert indicated that an expert rating of the app could also be interpreted as an evidence base.

"You could put the evidence base under the same umbrella with expert rating. An evidence base can also be at home, of users who say 'great'. Then it is already evidence based, without a theoretical foundation." (health science expert panel, expert in behavior change)

Fun

Some of the BCT's were described in detail. Fun, also called 'enjoyment', was described as a prerequisite for a good app. Nine out of eleven experts found this feature important for the effectiveness of physical activity-related apps (four in technology expert panel and five in technology expert panel). Fun could be achieved by using games in apps (i.e. gamification). According to one of the behavior change experts, having fun could increase intrinsic motivation of individual athletes.

"If people have fun. That corresponds to intrinsic motivation. 'This is fun; this is what I like to do, that should matter as well.' This could be achieved with gamification, but also with badges or something you can earn." (health science expert panel, expert in behavior change)

Another feature that experts from health science expert panel related to fun and gamification is engagement. Engagement means continued use of the app. Overall, experts thought fun, motivating elements, challenges, rewards and tailoring of the app may add to engagement. One expert formulated it as follows:

"If you really want to change behavior, people should use the app. That is why I think engagement is very important. Because you see that people use an app only once or twice. Also, in studies the app use reduces drastically. How can the user be engaged in such a way that he really gets what he needs to establish behavior change." (health science expert panel, expert in health sciences)

Social component

According to five experts from both panels, social components are important for the effectiveness of physical activity-related apps. Being a member of a community could help people to stimulate sport participation. Furthermore, the possibility of following others and sharing data may help. Whether your friends or professionals use the app as well as their app ratings may support use and effectiveness of apps as well. One expert explained the social component as:

"Who else uses the app? Is there a social component included? For instance, that you are able to follow others and others can follow you. That you can share specific statistics, that people can respond to." (health science expert panel, expert in injury prevention or monitoring)

Monitoring/statistics

Monitoring was another BCT that was described as an important feature that can contribute to the effectiveness of physical activity-related apps. Five experts from health science expert panel thought that monitoring physical activity behavior was important for motivating people to be more active. In addition, feedback on that performance should be provided.

"In case of the app I use, you can get an overview of how much kilometers you cycled last month, what your mean speed was and where you have cycled. You could see that over the whole year and I think that is motivating." (*health science expert panel, expert in injury prevention or monitoring)*

Two experts thought that reliable measures were necessary for the ability of the app to monitor the behavior.

"Therefore, you need reliable measures. This is something else than technical problems. These measures need to be up-to-date, so timely synchronization is needed. And a meaningful representation is needed. This could be different for each person and can be graphs, tables, means, totals, etcetera." (health science expert panel, expert in persuasive technology)

Reward system

Another BCT was giving rewards. In other words, what do you get in return? Two experts (from each panel 1) highlighted that these rewards should be linked to goals or progression.

"That you receive a reward after reaching your goals. Even if it is a thumb up or something like that. In this case, the difficulty of the goal does not matter." (health science expert panel, expert in injury prevention and monitoring)

Feedback & coaching

Experts emphasized that a feedback option needs to be integrated in a physical activity-related app. Several feedback options were suggested. By some of them (technology expert panel), this was described as applied feedback and feedforward. These experts related feed forward to the ability of the app to anticipate, for instance predicting injuries based on the collected app data. Some suggestions were provided on the content of the feedback. In general, domain specific knowledge is required.

"Basically applied feedback includes knowledge of sports, motivational support and quality of coaching, and depends on the intended application." (technology expert panel, expert in Industrial Design)

In addition, a few experts from technology expert panel advised that a choice should be made between explicit or implicit feedback.

Furthermore, in health science expert panel a distinction was being made between motivational feedback and instructional feedback. One expert explained that motivational feedback should include positive framing and learning to cope with disappointments.

"You should be approached in a positive way, even if you haven't done anything that day. … Besides providing feedback on today was good, 'well done', or today was not that good so try harder, that you learn to cope with setbacks, that should be part of the game. Sometimes you have a bad day or a bad month, but that is okay. Even if you are highly motivated, sometimes it just goes badly." (health science expert panel, expert in persuasive technology)

Instructional feedback was described as instructions on how to execute tasks. At last, one expert health science expert panel thought a physical activity-related app should be able to motivate or challenge people, for instance by setting goals or helping you challenge yourself.

Coaching styles matter as well and should be tailored on the individual athlete.

"I think that some people just need a strict coach, while others need a nice coach. The tone of voice and the way of framing it (content, formulating messages) depends on the user. A smart app should take that into account." (health science expert panel, expert in behavior change)

Tailoring

Taking into account individual preferences and the context of users was seen as an important aspect that could improve the effectiveness of physical activity-related apps. The experts from both panels proposed several ways of applying tailoring. One is that the app is fit to the user or to everyday life. The app should match with the goals or context of the user. Furthermore, the app should fluctuate with someone's life. Personalization allows tailoring the app as well.

"Every form of personalizing, being that you could adapt your avatar or that your name is present in the app (on top)". (health science expert panel, expert in health sciences)

Tailoring could be applied at the moment a person starts using the app, but could also be continued during the whole process of using an app. Three experts from health science expert panel reported that the app should be aligned with the current level of health, knowledge, functioning, personal goals, level, competitiveness and personal characteristics, physical activity level and whether you are very inactive or active.

"That the app matches the current level of health, knowledge, functioning and personal goals. And this tailoring should continue along the process. To me, it is important that the tailoring should fluctuate with one's life." (health science expert panel, expert in behavior change)

"Competitiveness, I think this is important in relation to the level. One person is more competitive than the other. Especially in a highly inactive group, you see that these people are not focused on competition at all. While a large amount of currently available apps are focused on competition." (health science expert panel, expert in persuasive technology)

One expert from health science expert panel proposed that continued tailoring may not be necessary for factors such as personality or competitiveness, that do not change. It would be sufficient to measure such factors at the moment someone starts using the app. Another element of tailoring is the flexibility of the app, in other words being able to adjust the app and adaptivity of the app (rated by four experts from technology expert panel and one from health science expert panel). Adaptivity means that the app is able to cope with different types of users.

"For instance, if your running performance improves, the app should develop as well." (technology expert panel, expert in industrial design)

One step further would be that the app should anticipate on the user. For instance, by accounting for schedules and location. Seven experts (two from technology expert panel and five from health science expert panel) described this feature as context awareness. As an example, a recommender system was described. A recommender system is a machine-learning, information-retrieval software tool that predicts what a user may or may not like or need [48]. It can provide suggestions based on these predictions.

"That you reckon with someone's context. That it can account for the fact that not all things go as planned. In the sense of a daily or weekly pattern or exceptions like, today I will sit in my car all day, so leave me alone. The app should account for that." (health science expert panel, expert in persuasive technology)

(about recommender system) "Examines your behavior, maybe your friend's behavior, and how it develops. And says at the right time, isn't this something for you." (health science expert panel, expert in behavior change)

Health / safe

According to one of the experts from health science expert panel, a check on health before one starts using an app would contribute to the effectiveness of that app. Also learning to listen to your own body may help.

"Do you perceive small complaints. We notice that during running, people do not know how to cope with these small complaints. They just continue until they go too far. The app should alert the user on the occurrence of small complaints and should give advice based on that information. For instance, now it is better to take a walk or take the bike instead of going for a run." (health science expert panel, expert in injury prevention and monitoring)

The same expert mentioned the importance of providing general information about healthy behavior. Users need information about what healthy behavior is. For instance, how can they safely increase their activity? Information about rest, sleep, injuries and food intake could be integrated into the app as well. This way awareness about healthy behavior could be achieved.

2. Entry requirements

The second theme includes entry requirements of the app. These are minimum conditions that support the use of the app. Examples are looks and usability, image of the app and other requirements such as privacy and costs of the app.

Looks & usability

At first, form, language, design, tone and interaction were described as important entry requirements for an effective app (mostly technology expert panel). Second, usability was found important in both panels (one from technology expert panel and seven from health science expert panel) and was defined in several ways and was related to functioning and simplicity of the app.

"I see this as a precondition, as essential, if you design it badly, it won't work. Maybe, a precondition is to strongly formulated, because Pokémon Go was played as well, even though it did not function well. Servers that stop working, search functions that do not work, all signs of failure and even though people play the game." (technology expert panel, expert in ICT service-design)

"Does the app do what you expect from it and do specific functions work properly. It shouldn't be too complex and searching for functions should not take too much time." (health science expert panel, expert in injury prevention and monitoring)

One expert from technology expert panel related the usability to motivation to be active. The technical application and design of push notifications matter.

"Usability, or ease of use, does it motivate you? Think about a push notification if you haven't done a task. This is a more functional application to motivate you. Not so much the knowledge and content is important, but also the technical application of a push message." (technology expert panel, expert in ICT service-design)

Stability, reliability and robustness of the app were related to usability as well. One expert from panel 1 summarized it as follows.

"So actually it is about how much you trust the app." (*technology expert panel, expert in ICT service-design)*

"It means that it is stable, reliable and robust. Reliability is if you go for a run and it does not function anymore. So something essential is not working anymore. (technology expert panel, expert in ICT service-design)

A third requirement mentioned in technology expert panel is that the app should function properly, without bugs. Moreover, being able to connect the app to other tools (such as an online platform, activity tracker or smartwatch) or being able to exchange data between platforms (i.e. portability) contributes to the usability of the app.

"Connectivity means how many other tools can I connect to? Think about technical requirements, if I need an expensive smartwatch, that would be restrictive… Or when it is not available in a certain country or on a platform." (technology expert panel, expert in ICT service-design)

Image of app

In general, some experts from technology expert panel indicated that the quality of the features in the app was important. In addition, other experts from health science expert panel noticed that the image of the app may contribute to the effectiveness of an app. The image of the app depended on the visibility, exposure and popularity of the app. Exposure was described as brand awareness.

"If there are a thousand apps in the app stores, you should be able to look at a screen shot and know 'this is what I was looking for'. … This has to do with exposure and marketing, which is related to the use of other people, by peers and peer ratings." (health science expert panel, expert in behavior change)

Reliability may contribute to the image of the app as well (health science expert panel). One part of reliability was that the measurements and feedback should function properly. Reliability was described as credibility, do people believe the app works. In addition, credibility may depend on who has built the app.

"If the app says you should execute these exercises or walk a certain distance. As a critical citizen I would think 'what do you know about it'. If it is built and validated by scientists or a physiotherapist, I may be more willing to do it." (health science expert panel, expert in behavior change)

Other requirements

Two other entry requirements were discussed, costs of the app (technology expert panel and health science expert panel) and privacy (health science expert panel). Some experts thought that people would be more willing to download an app if it is free. However, according to some of the experts, you could see it as an investment as well. When you invest money in an app, then you may be more motivated to continue using it and potentially stay active as well.

"If you buy expensive running shoes, the chance is higher that you continue running, as you invested so much." (health science expert panel, expert in behavior change)

Two experts thought the price-quality ratio was more important for the effectiveness of an app, than the price only:

"The price does not determine the quality! That is not how I experience it." (*technology expert panel, expert in ICT service-design)*

Privacy was described as an upcoming topic. In other words, what do you have to say about your data, but also what do app owners do with the collected data of users. The experts indicated that knowing how the privacy of your data is secured is an important entry requirement.
