# Supplementary material for: Analysis of the Features Important for the Effectiveness of Physical Activity–Related Apps for Recreational Sports: Expert Panel Approach
Source: JMIR Mhealth Uhealth. 2018 Jun 18;6(6):e143. doi: 10.2196/mhealth.9459 (PMC6028765; doi:10.2196/mhealth.9459)
Supplement: Multimedia Appendix 5 [file mhealth_v6i6e143_app5.pdf]

Multimedia Appendix 5 Coding scheme

Theme	Subtheme	Features
Features related to (behavior change) theories	Theoretical or evidence base	
	Fun	Fun<br>Engagement
	Social component	Social<br>Peer rating and use
	Monitoring/statistics	
	Reward system	
	Feedback & coaching	Applied feedback & Forward<br>Motivational feedback<br>Instructional feedback<br>Coaching styles
	Tailoring	Fit to user<br>Tailoring<br>Flexibility/adjustable/adaptive<br>Anticipating
	Health / safe	Check on health<br>General information about healthy behavior<br>Sustainable training plan<br>Increase awareness
Entry requirements	Looks and usability	Look & feel<br>Usability<br>Stability<br>Function properly<br>Connectivity<br>Portability
	Image	Visibility / exposure / reputation<br>Reliability<br>Qualitative Features / Content
	Other requirements	Costs<br>privacy
